# Priming of Defense Systems and Upregulation of *MYC2* and *JAZ1* Genes after *Botrytis cinerea* Inoculation in Methyl Jasmonate-Treated Strawberry Fruits

**DOI:** 10.3390/plants9040447

**Published:** 2020-04-02

**Authors:** Felipe Valenzuela-Riffo, Paz E. Zúñiga, Luis Morales-Quintana, Mauricio Lolas, Marcela Cáceres, Carlos R. Figueroa

**Affiliations:** 1 Institute of Biological Sciences, Campus Talca, Universidad de Talca, Talca 3465548, Chile; felvalenzuela@utalca.cl (F.V.-R.); paz.zuniga@utalca.cl (P.E.Z.); 2 Multidisciplinary Agroindustry Research Laboratory, Instituto de Ciencias Biomédicas, Universidad Autónoma de Chile, Talca 3467987, Chile; luis.morales@uautonoma.cl; 3 Fruit Pathology, Faculty of Agricultural Sciences, Campus Talca, Universidad de Talca, Talca 3465548, Chile; mlolas@utalca.cl (M.L.); mcaceres@utalca.cl (M.C.)

**Keywords:** jasmonate pathway, methyl jasmonate applications, defense genes, *Fragaria* × *ananassa*, anthocyanin, lignin, gray mold, necrotrophic fungi

## Abstract

Several attempts have been made to study the effects of methyl jasmonate (MeJA) on plants in the past years. However, the comparative effects of the number and phenological time of MeJA applications on the activation of defense systems is currently unknown in strawberries. In the present research, we performed three field treatments during strawberry (*Fragaria* × *ananassa* ‘Camarosa’) fruit development and ripening which consisted of differential MeJA applications at flowering (M3), and the large green (M2 and M3) and red ripe (M1, M2, and M3) fruit stages. We also checked changes in gene expression related to plant defense against *Botrytis cinerea* inoculation post-harvest. In M3 treatment, we observed an upregulation of the anthocyanin and lignin contents and the defense-related genes, encoding for chitinases, β-1,3-glucanases and polygalacturonase-inhibiting proteins, after harvest (0 hpi), along with the jasmonate signaling-related genes *FaMYC2* and *FaJAZ1* at 48 h after *B. cinerea* inoculation (48 hpi) during postharvest storage. Although we did not find differences in gray mold incidence between the MeJA treatments and control, these results suggest that preharvest MeJA treatment from the flowering stage onwards (M3) primes defense responses mediated by the upregulation of different defense-related genes and retains the upregulation of *MYC2* and *JAZ1* at 48 hpi.

## 1. Introduction

Crop plants constantly suffer from stress caused by abiotic and biotic factors, with infectious fungi being the most harmful biotic factors affecting crops [1]. Strawberry (*Fragaria* × *ananassa*) is a relevant fruit crop worldwide due to its extraordinary organoleptic characteristics [2,3]. However, during postharvest storage, this fruit has a high susceptibility to being infected by necrotrophic fungi [4]. Gray mold disease, caused by the necrotrophic fungus *Botrytis cinerea*, is one of the most important postharvest diseases, affecting more than 200 plant species [5]. Furthermore, gray mold is one of the most damaging strawberry diseases worldwide, with a detrimental economic impact on the strawberry fruit industry [5,6]. Postharvest necrotrophic fungi are controlled mainly by the application of synthetic fungicides and chemical products. However, consumer pressure has increased in favor of the reduced use of agrochemicals. In this sense, there is a scientific interest in studying natural compounds that could provide an alternative to chemical pesticides as has been reported for the containment of *B. cinerea*-associated infections [7]. One alternative is activating natural plant defense systems through the use of the plant hormone methyl jasmonate (MeJA), which belongs to the group jasmonates (JAs), and has been described as a defense-related molecule and a safety inductor of resistance during postharvest storage in many fruits [8,9,10,11,12,13,14]. Different studies have shown the beneficial effects of exogenous MeJA applications in fruits, such as the resulting higher levels of phenolic compounds, especially lignin and anthocyanins [13,15,16,17].

At the molecular level, the perception of *B. cinerea* and other necrotrophic pathogens activate different genes such as those encoding for pathogenesis-related proteins (PRs) [18]. The main PRs classes produce damage to the cell wall structure of plant pathogens. Among these proteins, two hydrolytic enzymes β-1,3-glucanases (BGs; PR2) and chitinases (CHIs; PR3, PR4, PR8, and PR11) degrade different cell wall-associated polysaccharides in several fungi [19]. Other proteins, called polygalacturonase-inhibiting proteins (PGIPs), are highly expressed after inoculation with *B. cinerea*, indicating a participation in strawberry defenses against gray mold [19,20].

In turn, JAs cooperate in activating defenses against necrotrophic fungi [21]. Recently, we described for the first time the main JA biosynthesis- and signaling-related components during strawberry fruit development at the genetic level such as the enzyme 12-oxophytodienoate reductase (*FaOPR3*), the transcription factor MYC2 (*FaMYC2*), and the repressor protein jasmonate ZIM-domain 1 (*FaJAZ1*) [22,23]. The response of those genes at fruit postharvest upon differential preharvest MeJA applications is currently unknown in strawberries.

Thus, we aimed to analyze and compare the effects of MeJA applications at different phenological stages of the strawberry on the disease incidence, anthocyanin and lignin accumulation, and the expression levels of defense- and JA pathway-related genes during postharvest times. This is important to determine if MeJA applications performed during all the reproductive development period lead to a greater strengthening of the overall defense system than a single application at the ripe fruit stage. We found that preharvest MeJA applications performed from the flowering stage onwards led to the activation of fruit defense systems.

## 2. Results and Discussion

### 2.1. Response of MeJA-Treated Fruit to Infection Caused by Botrytis cinerea and Other Postharvest Fungal Pathogens

**Figure 1:**
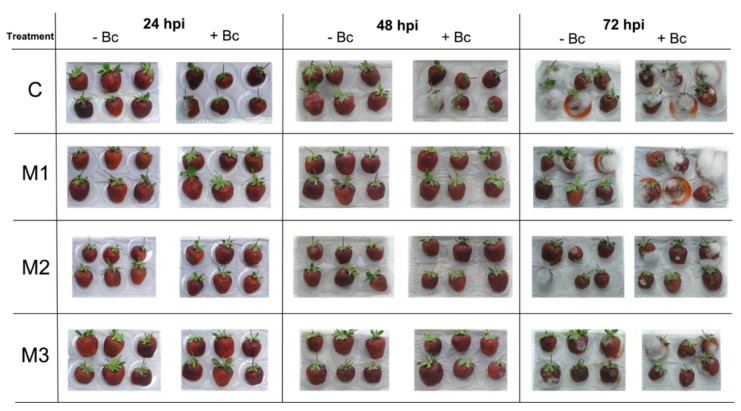
Evolution of postharvest necrotrophic fungi (including gray mold) incidence on representative strawberry (*Fragaria* × *ananassa* ‘Camarosa’) fruits belonging to the different treatments performed in the present research. Each group of fruits subjected to different methyl jasmonate (MeJA) treatments during fruit development (C, M1, M2, and M3) were inoculated by injecting 20 μL of the *Botrytis cinerea* conidia suspension (+Bc) or distilled water (−Bc) with a sterile syringe. Fruits were maintained in humidity chamber at 20 °C up to 72 h post-inoculation (hpi). Gray mold incidence in +Bc and −Bc fruits was recorded at 24, 48 and 72 hpi. For experimental details, see the Materials and Methods section.

With the aim to contrast the response time of the methyl jasmonate (MeJA) applications during strawberry development, we performed from one to three applications at different developmental stages of strawberries grown in field conditions. Infections with *B. cinerea* could occur from early stages in open flowers to late infections by fungal penetration in receptacle tissue [6]. Therefore, we applied MeJA three times at the flowering (F), large green fruit (LG), and 100% red ripe fruit (RR) stages (M3 treatment); two applications at the LG and RR stages (M2 treatment), and one application at RR stage (M1 treatment). After the last application, fruits were harvested, transported to the laboratory, and then inoculated with *B. cinerea* conidia (+Bc) or water (−Bc). The progression of infection was observed for 72 h post-inoculation (Figure 1, Table 1).

We did not observe statistically significant differences of gray mold incidence values (%) between the different treatments (Table 1). This could be because the possible defense-related effects triggered by MeJA are not enough to counteract the progression of the infection due to facts such as the possible high aggressiveness of the *B. cinerea* strain and the conidia inoculation method used in the present research. However, at different hours post-inoculation, we observed that M3 treatment presented null incidence values up to 48 hpi for −Bc fruits (Table 1, Figure 1). A reduction of the incidence level during postharvest storage was reported in *Fragaria chiloensis* fruits that were also inoculated with *B. cinerea* conidia and treated preharvest with three MeJA applications [11]. It is important to note that incidence in −Bc fruit probably reflects the appearance of systemic Botrytis infection commonly found in strawberry [24]. The null incidence found in M3 treatment at 24 and 48 hpi in −Bc fruits could imply the relevance of applying MeJA from F stage onwards. In this sense, protecting flowers of *B. cinerea* infections seems to be relevant to reduce postharvest gray mold incidence, as has been reported previously [25].

Additionally, we analyzed the incidence of other typical postharvest rot-associated fungi of strawberry fruit such as *Penicillium* sp., *Rhizopus* sp., and *Cladosporium* sp. at 72 hpi (Table S1). We found a significantly lower incidence (0%) of *Penicillium* sp. in all MeJA treatments in comparison with control (16.7%), but not for *Rhizopus* sp. and *Cladosporium* sp. In this sense, Jin et al. [9] reported significantly lower incidence values of *Penicillium expansum* and *Rhizopus stolonifer* at the postharvest of peach fruit treated with MeJA at harvest. Although we did not perform an in vitro test to rule out a direct effect of MeJA on *Penicillium* sp., previous work indicated that MeJA does not alter either the spore germination or the germ tube length of *Penicillium citrinum* [26]. Future work in this regard would be necessary to clarify the mechanism of inhibition of *Penicillium* sp. incidence by MeJA in strawberry.

Despite the fact no differences were found in incidence levels of +Bc fruits, the results suggest that preharvest MeJA applications in strawberry decrease the infection progression of *B. cinerea* at postharvest depending on the developmental stage when they are applied, with M3 being the treatment which presented null gray mold incidence during the postharvest storage of −Bc fruits, suggesting that MeJA application from F stage onwards could help to decrease the systemic Botrytis infection. MeJA treatment also reduces gray mold disease in other fruits during postharvest, such as in peach [9] and grape [10].

### 2.2. Anthocyanin and Lignin Accumulation and Upregulation of Jasmonate- and Defense-Related Genes in MeJA Treated Fruit

**Figure 2:**
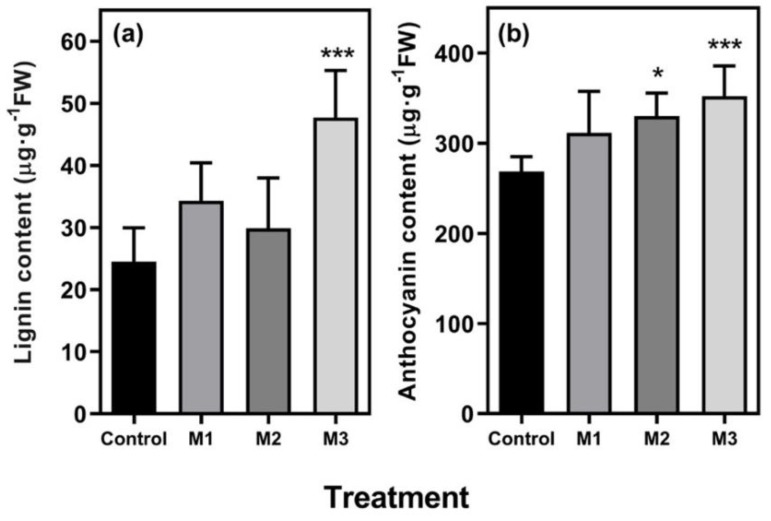
Lignin and anthocyanin contents of different methyl jasmonate (MeJA)-treated strawberry (*Fragaria* × *ananassa* ‘Camarosa’) fruit at postharvest (0 hpi). Changes in (**a**) lignin and (**b**) anthocyanin contents in fruit subjected to different methyl jasmonate treatments during fruit development (C, M1, M2, and M3) and analyzed postharvest. Data were analyzed by ANOVA test, and differences between means ± standard error (SE) (*n* = 3) were determined using the LSD test. Asterisks indicate significant differences between treatments and control (* *p* < 0.05; *** *p* < 0.001). For experimental details, see the Materials and Methods section.

To understand to some extent, the physiological and molecular mechanism underlying the defense response of MeJA-treated fruit, we analyzed changes in lignin and anthocyanin contents and defense-related gene expression in the harvested treated fruit. According to literature, MeJA treatment not only upregulated defense-related gene expression but also promoted the secondary metabolism, particularly through the activation of the phenylpropanoid pathway [16,27,28]. In this sense, we observed that M3 treatment promoted both a higher level of anthocyanin and lignin accumulation with respect to control fruits at postharvest (0 hpi) (Figure 2). The M2 treatment also increased the anthocyanin content regarding to the control treatment (Figure 2b). These results suggest that MeJA applications from the F or LG stages onwards are decisive for triggering the anthocyanin accumulation observed in 0 hpi (Table 2), as has also been observed in *F. chiloensis* [17]. With respect to lignin accumulation, we observed that M3 treatment results in the highest level, suggesting that MeJA application from the F stage onwards is conducive to lignin accumulation. The non-existence of differences between control and M1 treatments either in lignin or anthocyanin contents could be related with the time between the last application (at RR stage) and the laboratory analysis (the day after) would be very short to expect some stimulatory effect of MeJA on these parameters. In this sense, an increase in lignin and anthocyanin accumulation was observed at 2 and 5 days, respectively, in *Fragaria* species fruits under constant MeJA application [16,22].

**Figure 3:**
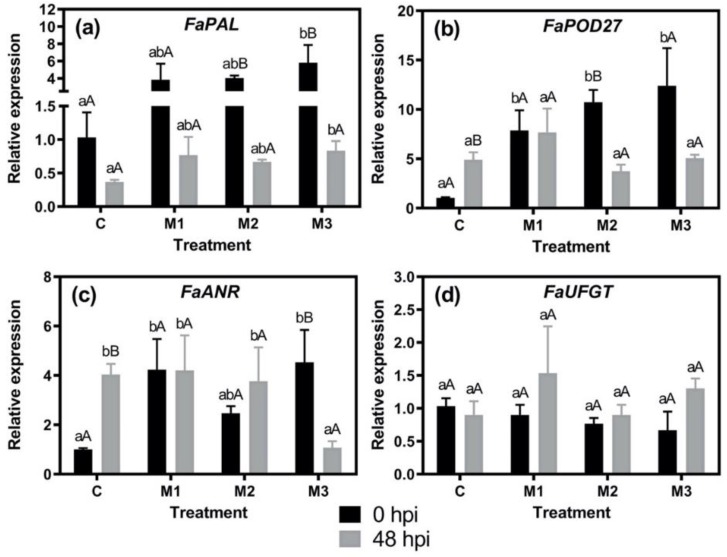
Expression profiles of genes encoding phenylpropanoid pathway-related enzymes of different methyl jasmonate (MeJA)-treated strawberry (*Fragaria* × *ananassa* ‘Camarosa’) fruit at 0 and 48 h post inoculation (hpi) with *Botrytis cinerea* conidia (+Bc fruits). Changes in the relative expression of (**a**) *FaPAL*, (**b**) *FaPOD27*, (**c**) *FaANR*, and (**d**) *FaUFGT* in fruit subjected to different methyl jasmonate treatments (C, M1, M2, and M3) and analyzed at 0 and 48 hpi. Differences between means ± standard error (SE) (*n* = 6) were determined by ANOVA and LSD test (*p* < 0.05). Different capital letters indicate significant differences between 0 and 48 hpi on each treatment. Different lower-case letters indicate significant differences between treatments on each time (0 or 48 hpi). For experimental details, see the Materials and Methods section.

The analysis of gene expression in response to MeJA applications through postharvest time was performed in +Bc fruits at 0 and 48 hpi. We selected these time points due to the fact that at 0 hpi, fruits are not infected with the fungus (i.e., 0% incidence) and 48 hpi represented an average time of the gray mold progression (Table 1) according to a previous report [17]. Then, we investigated the associated gene expression changes for anthocyanin and lignin biosynthesis, observing that the expression of *FaPOD27* showed an upregulation in all MeJA treatments at 0 hpi (Figure 3b), whereas the expression levels of *FaUFGT* in MeJA treatments did not change regarding control (Figure 3d). In turn, the expression of *FaPAL* and *FaANR* showed an upregulation in M3 treatment in relation to control at 0 hpi (Figure 3a,c) which was associated with the highest level of anthocyanin observed (Figure 2b). Noticeably, the pattern of *FaPOD27* at 0 hpi (Figure 3b) was closely related to the lignin accumulation profile (Figure 2a). In this sense, Concha et al. [16] demonstrated a high upregulation of *POD27* under MeJA treatment during the ripening of *F. chiloensis* fruit under laboratory conditions, which strongly agrees with the lignin accumulation profile observed in the same treated fruits. In the M1 treatment, the absence of correlation between the *FaPOD27* and *FaANR* expression levels with the lignin and anthocyanin accumulation patterns at 0 hpi, respectively, may be due to the participation of other genes or gene family members belonging to the phenylpropanoid pathway that we did not analyze. It has been shown that MeJA treatment upregulates most of the phenylpropanoid pathway-related genes in *F. chiloensis* fruits [16], and the existence of several gene family members for anthocyanin biosynthesis has been evaluated in *Fragaria* species [27].

**Figure 4:**
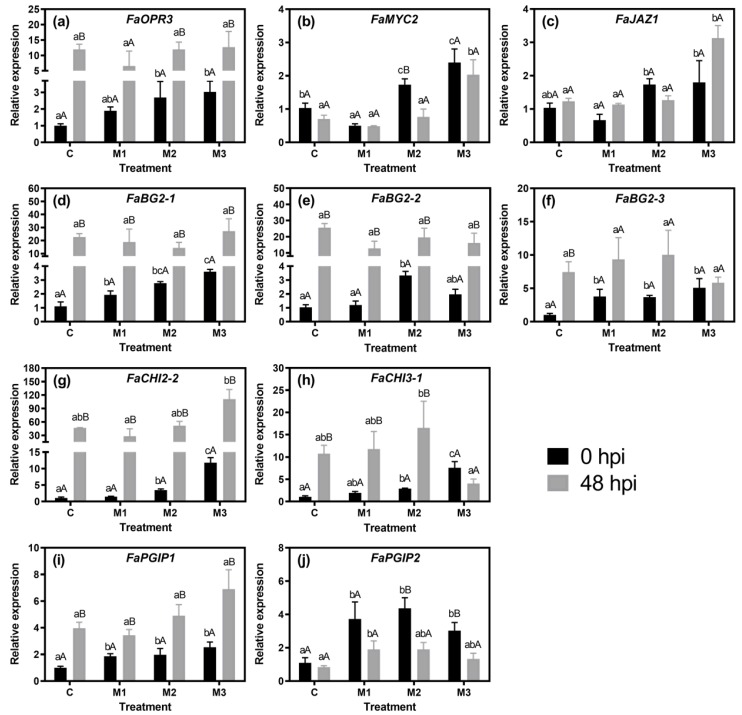
Expression profiles of genes encoding for jasmonate pathway-related components, PR- (β-1,3-glucanases and chitinases), and PGIP-related genes: (**a**) *FaOPR3*, (**b**) *FaMYC2*, (**c**) *FaJAZ1*, (**d**) *FaBG2-1*, (**e**) *FaBG2-2*, (**f**) *FaBG2-3*, (**g**) *FaCHI2-2*, (**h**) *FaCHI3-1*, (**i**) *FaPGIP1*, and (**j**) *FaPGIP2* in strawberry (*Fragaria* × *ananassa* ‘Camarosa’) fruit subjected to different methyl jasmonate treatments (C, M1, M2, and M3) and analyzed at 0 and 48 h post-inoculation (hpi) with *Botrytis cinerea* conidia (+Bc fruits). Differences between means ± standard error (SE) (*n* = 6) were determined by ANOVA and LSD test (*p* < 0.05). Different capital letters indicate significant differences between 0 and 48 hpi on each treatment. Different lower-case letters indicate significant differences between treatments on each time (0 or 48 hpi). For experimental details, see the Materials and Methods section.

Concerning the analysis of the changes in defense- and jasmonate (JA) pathway-related gene expression, an overall upregulation in JA biosynthesis- (*FaOPR3*), PR- (*FaBG1-2*, *FaBG2-2*, *FaCHI2-2*, and *FaCHI3-1*), and PGIP- (*FaPGIP1*) related genes was observed in both the control and MeJA treatments in response to pathogen infection (48 h-post-inoculation) (Figure 4a,d,e,g–i). Concurrently, *FaMYC2*, *FaJAZ1*, *FaBG2-3*, and *FaPGIP2* showed no significant changes for all treatments (Figure 4b,c,f,j), with the exception of an upregulation for *FaBG2-3* in the control treatment (Figure 4f), and a downregulation for *FaMYC2* and *FaPGIP2* in the M2, and M2 and M3 treatments, respectively (Figure 4b,j).

Notably, jasmonates (JAs) have been associated with defense mechanisms to different stresses, including that related to necrotrophic fungi such as *B. cinerea* [21]. The enzyme OPR3, the transcription factor MYC2, and JAZ proteins are key components for JA synthesis and signaling pathways, respectively [29,30]. In the present research, *FaOPR3* was upregulated by M2 and M3 treatments at 0 hpi in the control, and an upregulation over 10-fold was observed in the control, M2 and M3 treatments upon pathogen infection (48 hpi) (Figure 4a). In the case of *FaMYC2*, an upregulation was observed in the M2 and M3 treatments at 0 hpi (Figure 4b). Thus, MeJA treatments from the F or LG fruit stages onwards would have a priming effect on both *FaOPR3* and *FaMYC2*. An upregulation upon MeJA treatment has also been shown for *OPR3* and *MYC2* in both *F*. *chiloensis* and *F*. × *ananassa* fruit under laboratory conditions [16,23]. In the case of *FaJAZ1*, our previous work showed a transient gene expression at 30 min after MeJA supply in an in vitro ripening assay [23], which agreed with the no priming effect observed for the *FaJAZ1* expression in the present research. Moreover, *FaMYC2* and *FaJAZ1* were upregulated at 48 hpi in the M3 treatment (Figure 4b,c), which indicates that a combination effect is produced by the presence of *B. cinerea* and the MeJA application (M3 treatment). Our results suggest that MeJA applications from the F or LG stages onwards (M2 and M3) activate JA-biosynthesis (*FaOPR3*) and -signaling (*FaMYC2*) through a priming effect (0 hpi), and in the case of MeJA applications from the F stage onwards (M3) the JA-signaling (i.e., the upregulation of *FaMYC2* and *FaJAZ1*) is switched on after *B. cinerea* inoculation (i.e., at 48 hpi) (Table 2). The role of *FaMYC2* could be crucial to mediate MeJA responses in fruits. In this sense, it was recently reported in tomato fruit, MeJA treatment induces *SlMYC2* expression and the beneficial effects of MeJA, such as chilling tolerance [31].

Regarding defense-associated genes, an upregulation in all of the PR- and PGIP-related genes analyzed (*FaBG1-2*, *FaBG2-2*, *FaBG2-3*, *FaCHI2-1*, *FaCHI3-1*, *FaPGIP1*, and *FaPGIP2*) was remarkably noted in MeJA treatments with respect to control at 0 hpi (Figure 4d–j, Table 2). Previously, the upregulation of these genes was also reported in an experiment with three preharvest MeJA applications in *F. chiloensis* fruit [11]. In that work, *FcBG2-1*, *FcBG2-3*, *FcPGIP1*, *FcPGIP2*, *FcCHI2-2*, and *FcCHI3-1* genes were upregulated in MeJA-treated fruits before the challenging of the pathogen. In the present research, the gene encoding for β-1,3-glucanase (*BG2-1*) and the two for chitinase (*CHI2-2* and *CHI3-1*) showed a similar expression pattern at 0 hpi, with an increased expression level as the fruit received more than one differential MeJA applications during the fruit development period (i.e., the M2 and M3 treatments) (Figure 4d,g,h). MeJA also induced the expression of a gene encoding for a grape chitinase [10]. In the case of *FaBG2-2*, the highest expression was observed in M2 treatment at 0 hpi (Figure 4e). The expression levels for *PGIP*s and *FaBG2-3* showed no differences between the different MeJA treatments, although all presented higher values in comparison with control at 0 hpi (Figure 4f,i,j). Overall, the possible MeJA priming effect observed for all defense-related genes analyzed (Table 2) does not correspond with the gray mold incidences values of MeJA-treated fruits with respect to control, since no statistically significant differences were observed (Table 1). The aggressiveness of the *B. cinerea* strain and the inoculation method used, along with the existence of the systemic Botrytis infection in fruits, could explain the low or null defense triggered by MeJA observed in the treated fruits.

On the contrary, the expression levels for all defense-related genes analyzed at 48 hpi seem to be independent of MeJA treatment but pathogen-dependent since they were similar to those presented by control (Figure 4d–j, Table 2)—with the exception of *FaPGIP2*, of which expression in the M1 treatment was higher than in the control (Figure 4j). Remarkably, the higher expression levels observed for *FaBG2-1*, *FaBG2-2*, and *FaCHI2-2*—both in the control and MeJA treatments at 48 hpi in relation to 0 hpi (Figure 4d,e,g)—were also observed previously for the orthologs *BG2-1*, *BG2-2*, and *CHI2-2* genes in *F. chiloensis* fruit at 48 h post-inoculation with *B. cinerea* having been treated with MeJA in preharvest [11]. In this sense, in spite of the differences in ripening-associated characteristics such as fruit softening and color acquisition between *F*. × *ananassa* and *F. chiloensis* [2,32,33], the defense mechanisms associated with chitinases and β-1,3-glucanases seem to be conserved between both *Fragaria* species. Overall for the PR and PGIP-related genes, we observed that an increasing number of differential MeJA applications upregulates the defense-related genes *FaBG2-1*, *FaCHI2-2*, and *FaCHI3-1* at 0 hpi (Figure 4d,g,h), which suggests that the MeJA application at flowering stage is relevant for a higher upregulation of the expression of those genes. It is important to point out that under the current experimental conditions, the upregulation pattern for *FaOPR3*, *FaBG2-1*, *FaBG2-2*, *FaBG2-3*, *FaCHI2-2*, *FaCHI3-1*, and *FaPGIP1* at 48 hpi in regard to 0 hpi suggests that it is dependent of *B. cinerea* inoculation and independent of MeJA effects, since no differences were observed with the control (Figure 4a,d–i, Table 2). An exception to this observation was *FaPGIP2*, which maintained its upregulation in the M1 treatment 48 h after pathogen inoculation (Figure 4j).

In summary, for defense-related genes, we observed an upregulation in response to MeJA applications (0 hpi), indicating a possible priming effect by MeJA for all defense-related genes analyzed, including those related with anthocyanin and lignin biosynthesis, and JA biosynthesis and signaling (Figure 1, Figure 2 and Figure 3, Table 2).

## 3. Materials and Methods

### 3.1. Plant Material and Hormone Treatments

Strawberry (*Fragaria* × *ananassa* ‘Camarosa’) fruits analyzed in the present research were harvested from methyl jasmonate (MeJA)-treated plants grown in a commercial plantation established in Pelluhue, Maule Region, Chile (latitude 35°47′ S; longitude 72°33′ W). Approximately 200 strawberry plants were divided into twelve plots under the same agronomic management (without application of fungicides). Flowers were marked at the beginning of the experiment (with the aim to analyze the same biological material at the end of the assay), and three MeJA (M1, M2, and M3) and the control (C) treatments were established. Each treatment corresponds to three aleatory plots. Specifically, M1, M2, and M3 treatments consisted of one, two, and three MeJA applications, respectively, at different fruit developmental stages, by spraying 250 μmol L^−1^ MeJA (Sigma-Aldrich, St. Louis, MO, USA) [17] each time as follows: at flowering (F) (M3 treatment), large green fruit (LG) (after 27 days of the application at flowering; M3 and M2 treatments), and 100% red ripe fruit (RR) (after 7 days of the application at large green stage; M3, M2, and M1 treatments) stages. C treatment consisted of the application of distilled water at the same time as the MeJA treatments. Then, fruits from treated plants were harvested at the RR stage on the same day, one hour from the last MeJA application. Then, these fruits were immediately transported to the laboratory under refrigerated conditions (approximately 2 h later). Fruits without surface damage were kept at 4 °C for 12 h and selected for further analyses based on uniformity in size, shape, and color.

### 3.2. Botrytis cinerea Inoculation in Fruits from Different Treatments

The *Botrytis cinerea* strain used in this research was isolated from infected strawberries (*F.* × *ananassa* ‘Albion’) grown in San Pedro, Metropolitan Region, Chile (latitude 33°52′ S; longitude 71°26′ W). Cultures were maintained in 2% (w/v) potato dextrose agar (PDA) plates at 20 °C. For inoculum production, PDA plates were incubated in a 12 h photoperiod at 22 °C. A diluted suspension of 10^4^ conidia ml^−1^ in distilled water was used for inoculation. Surfactant [0.02% (v/v) Tween-20] was used in the final suspension and inoculation control (distilled water). The following procedures were performed according to Saavedra et al. [11]. Selected ripe fruits were superficially disinfected by 1 min immersion in 1.5% (w/v) sodium hypochlorite, washed three times with distilled water, and the surface was air-dried in a laminar flow cabinet. Then, one group of fruits consisting of 18 fruits per treatment (three biological replicates corresponding to each plot of six fruits each) were inoculated by injecting 20 μL of the spore suspension (+Bc) or distilled water (−Bc) with a sterile syringe. Fruits were maintained in a humidity chamber at 20 °C up to 72 h-post-inoculation (hpi) (Figure 1). The incidence of gray mold in +Bc and −Bc fruits was recorded at 0, 24, 48, and 72 hpi and measured as the percentage of infected fruit for each treatment and time [11]. Incidence at 0 hpi (after 30 min of inoculation) was considered as 0, since the fruit was disinfected and no visual infection was observed at that time. Furthermore, the incidence of typical postharvest fungi such as *Penicillium* sp., *Rhizopus* sp., and *Cladosporium* sp. were evaluated after 72 hpi in −Bc fruits by phenotypes on fruits and then corroborated by medium-culturing. Finally, another group of fruits consisting of 36 +Bc fruits per treatment were used for sampling at 0 and 48 hpi (three biological replicates of six fruits each per two time points) for gene expression analyses.

### 3.3. Analysis of Anthocyanin and Lignin Contents

A pool of fruits at 0 hpi was used as a replicate for anthocyanin and lignin contents analyses. Anthocyanin content was quantified spectrophotometrically by the pH method [34] modified for strawberry fruit samples, as in Delgado et al. [27]. Briefly, a fruit receptacle (2.5 g) was homogenized in extraction solution [10 mL of absolute ethanol and 1.5 N HCl (85:15, v/v)], incubated overnight at 4 °C, and then centrifuged at 12,000 rpm for 10 min at 4 °C. The ethanolic phase was diluted (1:4) with two different buffers: pH 1 buffer (0.025 M KCl) and pH 4.5 buffer (0.4 M sodium acetate). The extraction solution with each pH buffer was used as the blanks. Subsequently, the absorbance was quantified at 516, 520, and 700 nm. The anthocyanin content was calculated using the molar extinction coefficient of pelargonidin-3-glucoside (ε = 31,620 M^−1^ cm^−1^) [35] and expressed as micrograms of pelargonidin-3-glucoside equivalent per gram of fresh weight (FW) (*n* = 3).

Lignin content analysis was carried out according to Yeh et al. [36] and Saavedra et al. [17]. Cell wall extracts were prepared from 250 mg of frozen strawberry fruit [37,38] and, subsequently, the lignin content was determined by thioglycolic-acid assay. Each sample was mixed in 750 μL of distilled water, 250 μL of 37% (v/v) HCl, and 100 μL of thioglycolic acid (Sigma-Aldrich, St. Louis, MO, USA) and incubated at 80 °C for 3 h. The next steps were based on Campbell and Ellis [39]. The insoluble lignin was dissolved in 1 mL of 1 M NaOH, and the absorbance was registered at 280 nm. The lignin content was calculated from a linear calibration curve (0–20 μg) using hydrolytic lignin (Sigma-Aldrich, St. Louis, MO, USA) as standard. The results were expressed as μg of lignin per g of FW (*n* = 3).

### 3.4. Gene Expression Analysis

RNA was isolated from the strawberry receptacle tissue around the inoculation site (approximately 1 cm^3^) using the CTAB method [40] with modifications [11]. Three independent RNA extractions were carried out from each frozen pool of samples from +Bc fruit. High-quality RNA without genomic DNA contamination was used for cDNA synthesis using a ‘First Strand cDNA Synthesis Kit’ (Fermentas Life Science, MD, USA) as previously reported by Delgado et al. [27].

The mRNA abundance of the genes encoding for JA pathway-related components (12-oxophytodienoate reductase, *FaOPR3*; MYC2, *FaMYC2*; and jasmonate ZIM-domain 1, *FaJAZ1*), pathogenesis-related (PR) proteins β-1,3-glucanases (*FaBG2-1*, *FaBG2-2* and *FaBG2-3*) and chitinases (*FaCHI2-2* and *FaCHI3-1*), and polygalacturonase inhibiting proteins (*FaPGIP1* and *FaPGIP2*) was analyzed using quantitative reverse transcription PCR (RT-qPCR). Furthermore, the expression levels of lignin and anthocyanin biosynthesis-related genes were analyzed, including those encoding for phenylalanine ammonia lyase (*FaPAL*), peroxidase 27 (*FaPOD27*), anthocyanidin reductase (*FaANR*), and UDP glucose:flavonoid 3-O-glucosyl transferase (*FaUFGT*). Primers used for RT-qPCR analysis are recorded in Table S2. RT-qPCR was performed using the SYBR FAST qPCR kit (KAPA Biosystems, Boston, MA, USA) in a real-time PCR System (PikoReal, Thermo Fisher Scientific, Waltham, MA, USA) with a thermal profile previously reported [26]. The efficiency for each primer pair was checked using a cDNA from fruit samples as a template. Each reaction was performed in triplicate (technical replicate) and normalized against the expression level of glyceraldehyde-3-phosphate-dehydrogenase 1 (*FaGAPDH1*) gene. Relative expression was determined according to the 2^−∆∆Ct^ method [41] and expressed in arbitrary units. Control fruits of 0 were used as calibrators, designating them a nominal value of 1.

### 3.5. Statistical Analysis

The experiments were carried out using a completely randomized design. An analysis of variance (ANOVA) was used to compare the means of incidence and gene expression. Values of gene expression were normalized through log-transformation to compensate the non-normality of the data. A *p* ≤ 0.05 was considered statistically significant (LSD and Fisher tests). Statistical analysis was accomplished using the GraphPad Prism version 6.00 (GraphPad Software, San Diego, California, USA).

## 4. Conclusions

The present research addressed the response of strawberry (*Fragaria* × *ananassa* ‘Camarosa’) defense-related genes and those encoding for the key jasmonate (JA) signaling-related *MYC2* and *JAZ1* genes to methyl jasmonate (MeJA) applications in field conditions during different fruit developmental stages. We concluded that MeJA applications, most at flowering (F) or large green fruit (LG) stages onwards (M2 and M3 treatments, see Table 2), had a priming effect for defense systems including lignin and anthocyanin accumulation and the upregulation of the JA pathway-related (*FaOPR3* and *FaMYC2*), pathogenesis-related proteins (PRs), and polygalacturonase-inhibiting proteins (PGIPs)-encoding genes. This effect was only maintained for *FaMYC2* and *FaJAZ1* upon *B. cinerea* infection in the M3 treatment compared with the control and other MeJA treatments, suggesting that other defense-related mechanisms could be activated by JA signaling triggered by the fungus presence, and reinforced by MeJA treatments from the F stage onwards. Moreover, in the M3 treatment at 0 hpi, we found the highest expression of the genes encoding for chitinases, β-1,3-glucanase (*FaBG2-1*), *FaOPR3*, and *FaMYC2—*indicating that preharvest MeJA applications performed from the F stage onwards lead to the activation of fruit defense systems that could help to reduce the incidence of necrotrophic fungi during postharvest storage. In this sense, our next research interest is testing of the effects of MeJA application solely at the strawberry flowering stage.

## Figures and Tables

**Table 1:** Incidence (%) of gray mold in control (C), and methyl jasmonate (MeJA)-treated strawberry (*Fragaria* × *ananassa* ‘Camarosa’) fruits (M1, M2, M3 treatments) inoculated with *Botrytis cinerea* conidia (+Bc) or water (−Bc). For experimental details, see the Materials and Methods section.

Treatment	+Bc			−Bc		
	Hours Post-Inoculation (hpi)			Hours Post-Inoculation (hpi)		
	24	48	72	24	48	72
C	22.2 ± 7.9 ^1^	50 ± 27.2	55.6 ± 15.7	5.6 ± 7.9	22.2 ± 20.8	50 ± 27.2
M1	22.2 ± 20.8	50 ± 13.6	50 ± 13.6	5.6 ± 7.9	11.8 ± 15.7	27.8 ± 15.7
M2	22.2 ± 7.9	33.3 ± 13.6	38.9 ± 7.9	5.6 ± 7.9	11.8 ± 15.7	27.8 ± 20.8
M3	11.1 ± 7.9	27.8 ± 13.6	33.3 ± 13.6	0	0	11.1 ± 7.9
*Significance*	n.s. ^2^	n.s.	n.s.	n.s.	n.s.	n.s.

^1^ Standard deviation. ^2^ Non-significant according to Fisher’s test, *p* ≤ 0.05.

**Table 2:** Upregulation of specific plant physiological processes and gene expression in different methyl jasmonate (MeJA) treatments (M1, M2, and M3) and corresponding developmental stages (F, LG, RR) of strawberry (*Fragaria* × *ananassa* ‘Camarosa’) regarding control (C) treatment both in 0 and 48 hpi. F: flowering; LG: large green fruit; RR: 100% red ripe fruit. For experimental details, see the Materials and Methods section.

Upregulated Plant Physiological Processes and Genes	Priming Effect (0 hpi)	After *Botrytis cinerea* Inoculation (48 hpi)
Lignin and Anthocyanin Biosynthesis		
Lignin Content	M3 (from F stage onwards)	--- ^1^
Anthocyanin Content	M2, M3 (from F or LG stages onwards)	--- ^1^
*PAL*	M3 (from F stage onwards)	M3 (from F stage onwards)
*POD27*	M1, M2, M3 (at least at RR stage)	No differences ^2^
*ANR*	M1, M3 (from F stage onwards or at RR stage)	No differences ^2^
*UFGT*	No differences ^2^	No differences ^2^
Jasmonate Pathway-Related		
*OPR3*	M2, M3 (from F or LG stages onwards)	No differences ^2^
*MYC2*	M2, M3 (from F or LG stages onwards)	M3 (from F stage onwards)
*JAZ1*	No differences ^2^	M3 (from F stage onwards)
Defense-Related		
*BG2-1*	M1, M2, M3 (at least at RR stage)	No differences ^2^
*BG2-2*	M2 (from LG stage onwards)	No differences ^2^
*BG2-3*	M1, M2, M3 (at least at RR stage)	No differences ^2^
*CHI2-1*	M2, M3 (from F or LG stages onwards)	No differences ^2^
*CHI3-1*	M2, M3 (from F or LG stages onwards)	No differences ^2^
*PGIP1*	M1, M2, M3 (at least at RR stage)	No differences ^2^
*PGIP2*	M1, M2, M3 (at least at RR stage)	M1 (only at RR stage)

^1^ Measurement not performed. ^2^ No significant differences between MeJA treatments and control (*p* < 0.05).

## Supplementary Materials

The following are available online at https://www.mdpi.com/2223-7747/9/4/447/s1, Table S1. Incidence (%) of *Penicillium* sp., *Rhizopus* sp., and *Cladosporium* sp. in control (C)-, and methyl jasmonate (MeJA)-treated strawberry (*Fragaria* × *ananassa* ‘Camarosa’) fruits (M1, M2, M3 treatments) inoculated with water (−Bc) at 72 h of postharvest storage. For experimental details see the Materials and Methods section. Table S2. Primers sequences (5’→3’) used for quantitative reverse transcription PCR (RT-qPCR) analysis performed in the present research.
