# Supplementary material for: Priming of Defense Systems and Upregulation of MYC2 and JAZ1 Genes after Botrytis cinerea Inoculation in Methyl Jasmonate-Treated Strawberry Fruits
Source: Plants (Basel). 2020 Apr 2;9(4):447. doi: 10.3390/plants9040447 (PMC7238239; doi:10.3390/plants9040447)
Supplement: Supplementary file 1 [file plants-09-00447-s001.pdf]

Supplementary Material

Valenzuela-Riffo, Zúñiga et al.

Table 1. Incidence (%) of *Penicillium* sp., *Rhizopus* sp., and *Cladosporium* sp. in control (C)-, and methyl jasmonate (MeJA)-treated strawberry (*Fragaria* × *ananassa* 'Camarosa') fruits (M1, M2, M3 treatments) inoculated with water (–Bc) at 72 h of postharvest storage. For experimental details see the Materials and Methods section.

Treatment	Incidence (%)		
	***Penicillium* sp.**	***Rhizopus* sp.**	***Cladosporium* sp.**
C	16.7 a [1]	72.2 ± 7.9	0
M1	0 b	83.3 ± 13.6	11.1 ± 15.7
M2	0 b	77.8 ± 20.8	0
M3	0 b	88.9 ± 7.9	11.1 ± 7.9
Significance	<0.001	n.s. [2]	n.s.

[1] Different letters in the same column indicate significant differences between treatments (Fisher's test, $p \leq 0.05$). [2] Non-significant according to Fisher's test, $p \leq 0.05$.

Table 2. Primers sequences (5′→3′) used for quantitative reverse transcription PCR (RT-qPCR) analysis performed in the present research.

Target Gene	Primer Forward/Reverse	Reference
FaOPR3	5'- TCCACGGGACTCACCCTGTTCTCTC -3'<br>5'- TCGTTCAACGCTCGACACCTCGTC -3'	Garrido-Bigotes et al., 2018a
FaMYC2	5'- AGGGGATCCTGTCGTTTACC -3'<br>5'- TTCGGGGTCCACAACTCTAC -3'	Garrido-Bigotes et al., 2018b
FaJAZ1	5'- TGGGAGATCTGAACTCTGCTC -3'<br>5'- TTCCTCGGTTTCTCCATCAC -3'	Garrido-Bigotes et al., 2018b
FaCHI2-2	5'- GCACAACAGGTGATGTTGC -3'<br>5'- GTAATGACGTCGTGGCTTGA -3'	Saavedra et al., 2017
FaCHI3-1	5'- AGGTCTTCTTAGGCTCCCTGCGGC -3'<br>5'- CTTGGACCAAAGCATGACACCGCC -3'	Saavedra et al., 2017
FaBG2-1	5'- CTAAATATCTTCTTCCTGCCATA -3'<br>5'- AATGTTGTATCTATTGCTGTTG -3'	Saavedra et al., 2017
FaBG2-2	5'- ACCGGGACTCCCAAGAGACCAAATG -3'<br>5'- TGTGAGCCTGCACTAGCCAAAGGTG -3'	Saavedra et al., 2017
FaBG2-3	5'- TCCGAGAGTGGTTGGCCATCTGAAG -3'<br>5'- TCCATTTGGTCTCTTCGGAGTCCCG -3'	Saavedra et al., 2017
FaPGIP1	5'- TGCTATAATTCGATCTGTCCAAGG -3'<br>5'- ATTATCCAATTGGGTCAACTGCTC -3'	Saavedra et al., 2017
FaPGIP2	5'- TCCTCATGGAAATCCGACGCCGAC -3'<br>5'- ACCCTGTGAGATTGGGGAGCTTGCG -3'	Saavedra et al., 2017
FaPAL	5'- CAAGGGCGGCGATGCTAGTAAG -3'<br>5'- CCAAGTCACCCGACGACGAGAT -3'	Concha et al., 2013
FaPOD27	5'- ATTTCCATGATTGCTTTGTCAG -3'<br>5'- CAACGGCTAAGATGTCAGACC -3'	Concha et al., 2013
FaUFGT	5'- ATCGTGGCTTGACAAACAGAA -3'<br>5'- TGACCACAAGAATGGAACCCTA -3'	Delgado et al., 2018
FaANR	5'- GCTATCAGGTTCCATATCCATTACAC -3'<br>5'- TGAGCTCGGCAGACATCCT -3'	Delgado et al., 2018
FaGAPDH	5'- TTCATCACTACTGCCACCCAGAAGACTG -3'<br>5'- AGCAGGCAGAACCTTTCCGACAG -3'	Garrido-Bigotes et al., 2018a

References

1. Concha, C. M., Figueroa, N. E., Poblete, L. A., Oñate, F. A., Schwab, W., & Figueroa, C. R. (2013). Methyl jasmonate treatment induces changes in fruit ripening by modifying the expression of several ripening genes in Fragaria chiloensis fruit. Plant Physiol. Biochem. 70, 433–444. https://doi.org/10.1016/j.plaphy.2013.06.008
2. Delgado, L., Zúñiga, P., Figueroa, N., Pastene, E., Escobar-Sepúlveda, H., Figueroa, P., Garrido-Bigotes, A., Figueroa, C.R. (2018). Application of a JA-Ile Biosynthesis Inhibitor to Methyl Jasmonate-Treated Strawberry Fruit Induces Upregulation of Specific MBW Complex-Related Genes and Accumulation of Proanthocyanidins. Molecules 23, 1433. https://doi.org/10.3390/molecules23061433
3. Garrido-Bigotes, A., Figueroa, P. M., & Figueroa, C. R. (2018a). Jasmonate Metabolism and Its Relationship with Abscisic Acid During Strawberry Fruit Development and Ripening. J. Plant Growth Regul. 37, 101-13. https://doi.org/10.1007/s00344-017-9710-x
4. Garrido-Bigotes, A., Figueroa, N. E., Figueroa, P. M., & Figueroa, C. R. (2018b). Jasmonate signalling pathway in strawberry: Genome-wide identification, molecular characterization and expression of JAZs and MYCs during fruit development and ripening. Plos One. 13, e0197118. https://doi.org/10.1371/journal.pone.0197118
5. Saavedra, G. M., Sanfuentes, E., Figueroa, P. M., & Figueroa, C. R. (2017). Independent Preharvest Applications of Methyl Jasmonate and Chitosan Elicit Differential Upregulation of Defense-Related Genes with Reduced Incidence of Gray Mold Decay during Postharvest Storage of Fragaria chiloensis Fruit. Int. J. Mol. Sci. 18, 1420. https://doi.org/10.3390/ijms18071420
